# The Ethical War in Minneapolis

**Published:** 1893-06

**Authors:** 


### THE ETHICAL WAR IN MINNEAPOLIS.

In the number for March, 1891, we felt it to be a duty to ask the question, "Have we a Code of Ethics?" and in a recent article there was an effort to exhibit the attempts made to advertise, under many and specious disguises.

It has been apparent for a long period, with the self-respecting members of the dental profession, that this state of things would in a very short time result in a conflict of a more or less serious character.

That this was reserved for Minneapolis to commence is somewhat surprising, as it was generally supposed that this city stood pre-eminently as the exemplar in all that was elevated in professional training and ethical culture.

The interest which attaches to this should not be confined to the limit of any one district. It is a very broad question, and cannot be viewed upon any narrow or local basis.

The question whether the Faculty of the Department of Dentistry of the University of Minnesota was in fault is not so vital to the discussion as is that, To what extent can colleges extend their advertising? That colleges must advertise is certain. This is recognized in all professions, but conceding this, and without any special allusions to the one involved in the controversy, where is the line to be drawn between that which borders on the self-laudatory and egotistical and that recognized as proper and purely for the information of those directly interested? Circumstances must control the decision.

It is a satisfaction to know that there is a large number in at least one society in this country with the moral courage of its convictions, and in which a large minority—perhaps majority—are prepared to defend the ethics of the profession in their locality. If the same determined courage were manifested elsewhere, we would soon find associations, colleges, and individuals ceasing to advertise under the guise of an interview or by any of the questionable methods so common in medical and dental circles, for to do so would mean loss of standing in the profession and community.

While dentists generally cannot enter into the merits or demerits of a local controversy, they can understand the basic principles which underlie this conflict, and should extend a moral support to those who are endeavoring to elevate the work of our calling. To accomplish this there can be no divided interests and no border lines.

